# RETRACTION: Qi‐Dong‐Huo‐Xue‐Yin Inhibits Inflammation in Acute Lung Injury in Mice via Toll‐Like Receptor 4/Caveolin‐1 Signaling

**DOI:** 10.1155/ecam/9801813

**Published:** 2026-02-10

**Authors:** Evidence-Based Complementary Alternative Medicine

RETRACTION: L.‐Y. Xu, W.‐R. Cai, C.‐F. Ma, et al., Inhibits Inflammation in Acute Lung Injury in Mice via Toll‐Like Receptor 4/Caveolin‐1 Signaling,” *Evidence-Based Complementary and Alternative Medicine* 2018, 2018, 2373609, https://doi.org/10.1155/2018/2373609

The above article, published online on 11 February 2018 in Wiley Online Library (https://wileyonlinelibrary.com), has been retracted by agreement by John Wiley & Sons Ltd. The retraction has been agreed due to errors identified in Figure 5, specifically:

• The normal control panel in Figure 5a and the ALI panel in Figure 5c appear identical.
• The ALI + HDQ panel in Figure 5b shares unexpected, repeated elements with the Sham/ENaC‐α panel of Figure 4 in [1].

The authors were unresponsive to the concerns, and after assessment, the results are deemed unreliable. Therefore, the article is retracted.

The authors did not respond to the retraction.
